# Supplementary material for: Examining the Psychoactive Differences between Kratom Strains
Source: Int J Environ Res Public Health. 2023 Jul 21;20(14):6425. doi: 10.3390/ijerph20146425 (PMC10379209; doi:10.3390/ijerph20146425)
Supplement: Supplementary file 1 [file ijerph-20-06425-s001.zip › ijerph-2472345-supplementary.pdf]

Appendix A: Qualtrics Survey

Start of Block: Informed Consent Form

Department of Neuropsychopharmacology and Psychopharmacology

Purpose:

We are researchers at the department of Psychopharmacology, in Maastricht University, and we would like to invite you to participate in an online study about the use and effects of kratom. This survey is part of a larger study at Maastricht University that aims to gain insight into the use and effects of novel psychoactive substances, including novel opioids and novel psychedelics. In recent years, there has been an increased interest in novel opioid substances, including herbal substances such as kratom. However, not much is currently known about how and why people consume kratom, and what its effects are. Therefore, we would like to investigate this by means of this survey.

Selection of participants:

If you have ever consumed kratom, we invite you to participate in this study.

Voluntary Participation and time to decide:

This page is meant to inform you on your participation in this study. Participation in this study is completely voluntary. You have the right to decline to participate, and you can stop the survey and

withdraw from the study at any time without providing a reason, and without any negative consequences.

Procedure:

If you decide to participate in this study, you will be asked to fill out a questionnaire that will take approximately 10 - 20 minutes of your time, depending on your pace and depending on some of the answers you give.

The questionnaire will start with a few questions pertaining to your general health. Please note that you will be asked whether a healthcare professional has ever diagnosed you with a mental or physical condition, and whether you are currently taking any prescription medications. In the remainder of the questionnaire, you will be asked about your experience with kratom products, how and why you consume these and what type of effects you have experienced.

Risks and Benefits:

There are no direct risks or benefits associated with participating in this study.

Privacy: No personal data will be stored. Research data can be published and re-used in other research, but only in such a way that they cannot be traced back to you. This concerns the following data:

- Height
- Body weight
- Medication use
- Disease profile
- Smoking habits
- Alcohol consumption
- Age
- Gender
- Sex
- Level of Education
- Employment Status
- Marital Status
- Ethnicity
- Nationality
- Income

This research has been ethically reviewed and approved by the Ethical Committee of Psychology and Neuroscience (ERCPN), Maastricht University, 226_101_08_2020_A1. In case you decide to fill out the questionnaire, we ask that you take the questions seriously so that science can benefit from your experience with kratom. Of note, Maastricht University does not in any way encourage the use of any of the aforementioned substances, and excludes any responsibility for their use.

After reading this information page, we ask you to reflect on whether you would like to participate in this survey. If you have any additional questions, you can contact one of this study's researchers at any time using the contact information shown below:

Guido Huisman (graduate student)
Email: gr.huisman@student.maastrichtuniversity.nl
Phone:+13866272826

Natasha Mason (Principal Investigator)
Email: natasha.mason@maastrichtuniversity.nl

End of Block: Informed Consent

Start of Block: General Health

1 What is your height? (in feet and inches)

	0 1 2 3 4 5 6 7 8 9 10 11
ft.	
inches	

2 What is your body weight? (in lbs)

____

3 How often do you smoke/vape? (i.e. cigarettes or nicotine-containing e-liquids)

- ○ Never or rarely
- ○ Daily
- ○ At least once a week
- ○ Several times a week

4 How often do you consume alcohol?

- ○ Never or rarely
- ○ Daily
- ○ At least once a week
- ○ Several times a week

5 Are you currently taking one or more of the following types of prescription medications?

- [ ] Antidepressants (e.g. SSRI's, tricyclic antidepressants)
- [ ] Anxiety medication (benzodiazepines, e.g. Xanax)
- [ ] Antipsychotics (e.g. quetiapine, olanzapine, risperidone)
- [ ] Opioid pain killers (e.g. fentanyl, morphine, codeine)
- [ ] Stimulants (e.g. Ritalin, amphetamines etc)
- [ ] Others, namely: ____
- [x] I do not take any prescription medications

---

6 Has a physician/doctor/psychiatrist/nurse ever diagnosed you with one or more of the following conditions:

- [ ] Post Traumatic Stress Disorder (PTSD)
- [ ] Depression (major depressive disorder/persistent depressive disorder/dysthymia)
- [ ] Social Anxiety Disorder
- [ ] Generalized Anxiety Disorder
- [ ] Schizophrenia or other psychotic disorder
- [ ] Bipolar disorder

- [ ] Personality disorder
- [ ] Attention Deficit Disorder (ADD) / Attention Deficit Hyperactivity Disorder (ADHD)
- [ ] Addiction/substance use disorders
- [ ] Fibromyalgia
- [ ] Rheumatoid arthritis
- [ ] Chronic pain
- [ ] ⊗I have not been diagnosed with any of the above conditions

End of Block: General Health

Start of Block: Kratom color

7 Which color(s)/strain(s) of kratom do you generally consume?

- [ ] Green
- [ ] Red
- [ ] White
- [ ] Other: ____

End of Block: Kratom color

Start of Block: Kratom dosing

8a Which vendor or brand is your kratom?

- ○ Super Speciosa
- ○ Other ____

---

8 What form(s) of **${lm://Field/1}** product do you consume generally?

- ▢ Powder
- ▢ Tablets
- ▢ Capsules
- ▢ Tea

---

9 When you consume this color strain of kratom, what is the typical serving you take? Please indicate by dragging the slider(s) that apply to your consumption method(s).

	0 20
Gram	
Capsules	
Tablets	
Tea bags	
Teaspoons of powder	
Tablespoons of powder	

10 How often do you consume this color strain of kratom?

- ○ Several times per year or less
- ○ At least monthly
- ○ Several times per month
- ○ At least weekly
- ○ Several times per week
- ○ At least daily

○ Several times per day

○ Other: ____

11 How many servings of this color strain of kratom do you take in a typical week?

○ 0

○ 1 - 3

○ 4 - 7

○ 8 - 14

○ 15 - 21

○ 22 - 28

○ 29 - 36

○ 37 - 48

○ More than 48

12 How many servings of this color strain of kratom do you take on a typical day?

○ 0

○ 1

- 2 - 4
- 5 - 10
- 10 - 20
- More than 20

13 What time of day do you generally consume this color strain of kratom (multiple answers possible)

- Morning
- Afternoon
- Evening
- At night

14 When do you usually consume this color strain of kratom?

- Before a meal
- With a meal
- After a meal

Page Break

Start of Block: Ranking Motivations

24 Please rank your motivations for using this color strain of kratom

______ To treat a medical condition
______ To induce or enhance a spiritual experience
______ To feel less anxiety and/or stress
______ To help you relax or sleep
______ To feel elated, euphoric or intoxicated
______ To improve the quality of sex
______ To stay awake longer or to prolong a night out with friends
______ To lose weight or to reduce appetite
______ To be more sociable or to get more enjoyment out of social events
______ To help you concentrate, work or study
______ To improve the effects of other substances
______ To improve your mood or to feel less sadness/depression
______ Other (please specify)

Start of Block: VAS subjective drug effects

16

Please indicate to what extent you experience the effects mentioned below, when you consume this color strain of kratom. Try to think back on what you feel and observe during your kratom experiences, and how this compares to periods during which you are not taking kratom.

You can indicate this by clicking on the scales shown below and by dragging the blue slider towards the part of the scale that you deem to be appropriate for a given effect. In the scale, the left end (i.e. 0) represents the extreme of not experiencing the described effect at all, whereas the right end (i.e. 100) represents the extreme of experiencing the effect with great intensity.

	The effect is not present or not applicable to my situation	The effect is present to some degree	The effect is clearly present	The effect is present with great intensity
	0	50	100	
Feeling less physical pain	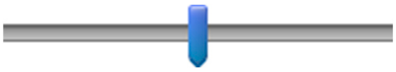			
Feeling happier	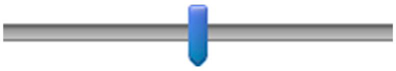			

Feeling more content	
Feeling more relaxed	
Feeling more nervous/tense	
Feeling calmer	
Feeling more on edge	
Being more easily agitated	
Having more mood swings	
Being better able to concentrate	
Being more easily distracted	
Feeling less depressed	
Feeling more anxious	
Being more forgetful	
Being less forgetful	
Feeling more energetic	
Feeling more fatigued	

Feeling more stimulated	
Feeling more nauseous	
Feeling more constipated than usually	
Vomiting more than usually	
Enjoying social events more than usually	
Being better able to stay up all night	
Being better able to fall asleep	
Having less insomnia	
Having more insomnia	
Feeling less sociable than usually	
Having more diarrhea than usually	
Having more stomachache than usually	
Feeling less withdrawal symptoms when withdrawing from other opioids (e.g. heroin, fentanyl etc.)	

Feeling less withdrawal symptoms when withdrawing from other substances that are not opioids (e.g. MDMA, cocaine, amphetamine, LSD, psilocybin)	
Feeling more socially withdrawn	
Feeling more sociable	
Not worrying as much	
Being less bothered by the actions of others	
Having an increased libido	
Having a decreased libido	
Experiencing more sexual satisfaction	
Having greater sexual dysfunction	

End of Block: Kratom dosing

Start of Block: General Demographic Information

17 What is your age?

- Under 18
- 18 - 24
- 25 - 34
- 35 - 44
- 45 - 54
- 55 - 64
- 65 or older

18 Which gender do you identify with?

- Male
- Female
- Non-binary
- Prefer not to say

19 What is your biological sex?

- Male
- Female

- ○ Other/Prefer not to say

---

20 Please select your highest completed level of education

- ○ Did not complete high school
- ○ High school graduate or equivalent
- ○ Some college (e.g. AA, AS or no degree)
- ○ Bachelor's degree (e.g. BA, BS, BSc, AB)
- ○ Advanced degree (e.g. MA, MS, MSc, MBA, PhD, MD)
- ○ Prefer not to say
- ○ Doctorate

---

21 What is your employment status?

- ○ Employed for wages
- ○ Employed - currently off sick
- ○ Out of work for less than 1 year
- ○ Out of work for 1 year or longer

- Homemaker
- Student
- Unable to work
- Retired
- Prefer not to say

22 What is your marital status?

- Married
- Widowed
- Divorced
- Separated
- Never married

23 How would you describe your ethnicity?

____

24 What is your nationality?

25 What is your total annual household income

- Less than $10,000
- $10,000 - $19,999
- $20,000 - $29,999
- $30,000 - $39,999
- $40,000 - $49,999
- $50,000 - $59,999
- $60,000 - $69,999
- $70,000 - $79,999
- $80,000 - $89,999
- $90,000 - $99,999
- $100,000 - $149,999
- More than $150,000
- Prefer not to say

End of Block: General Demographic Information
